# Effect of exercise on chronic neck pain and central sensitization: A protocol for a randomized crossover trial

**DOI:** 10.1113/EP091065

**Published:** 2023-03-29

**Authors:** Kexun Kenneth Chen, Mark Rowland Hutchinson, Paul Rolan, Rutger Marinus Johannes de Zoete

**Affiliations:** ^1^ School of Allied Health Science and Practice, Faculty of Health and Medical Sciences The University of Adelaide Adelaide South Australia Australia; ^2^ Adelaide Medical School, Faculty of Health and Medical Sciences The University of Adelaide Adelaide South Australia Australia; ^3^ Australian Research Council Centre of Excellence for Nanoscale Biophotonics The University of Adelaide Adelaide South Australia Australia

**Keywords:** central sensitization, chronic pain, exercise therapy, neck pain, physical exercise

## Abstract

Exercise‐induced hypoalgesia (EIH) has been found to vary widely within individuals with chronic neck pain (NP). Research has suggested that the presence of central sensitization within a subgroup of individuals with chronic NP might be a mediating factor to explain the relationship between exercise and improvements in patient‐reported outcomes. Furthermore, recent work has found that lactate might play a role in the development and maintenance of chronic pain. The immediate effect of a single bout of physical exercise on central sensitization in individuals with chronic NP and the relationship between lactate concentration, central sensitization and pain sensitivity are to be investigated. Eighty adult participants with chronic NP will be recruited for this randomized crossover trial. Outcome measures, including temporal summation, conditioned pain modulation, EIH and lactate concentration, will be assessed before and after low‐ and high‐intensity bicycling exercise. The outcomes of this study will provide new insights into the mechanistic effect of exercise on central sensitization in individuals with chronic NP and have the potential to add important information to the current exercise prescription guidelines for individuals with chronic NP. This study has been approved by the Human Research Ethics Committee, The University of Adelaide (H‐2022‐082) and registered in the Australian New Zealand Clinical Trials Registry (ACTRN12622000642785p).

## 1 INTRODUCTION

Neck pain (NP) is one of the leading chronic musculoskeletal pain conditions, with one of the highest years‐lived‐with‐disability (YLD) and leading causes of disability (Vos et al., 2016), and YLD has increased by 29% between 1990 and 2010 (Hoy et al., 2014). The majority of the global population, ≤70%, experiences NP at least once in their lives (Safiri et al., 2020). Of this population, 50–85% may not have complete remission of symptoms and may develop chronic NP (Safiri et al., 2020).

Multiple modalities of rehabilitation, such as physical exercise, psychology, manual therapy and acupuncture, have been used as treatment for chronic NP. Among these modalities of treatment, exercise has been found to be the best evidence‐based intervention and has been an integral component of the multimodal care plan in clinical guidelines for treatment of chronic NP (Sterling et al., 2019). However, current evidence for the effectiveness of exercise interventions for improvement of pain intensity and disability of chronic NP is modest at best (de Zoete, Armfield, et al., 2020; Price et al., 2020; Sterling et al., 2019). Central sensitization (CS) has been suggested to be a mediating factor to explain the relationship between exercise and improvements in patient‐reported outcomes (Ellingson et al., 2016; Jull et al., 2007) and has been found to be present in a subgroup of chronic musculoskeletal pain populations (Kim et al., 2015; Nijs et al., 2010; Sterling et al., 2006). A recent systematic review found that exercise might alter functional characteristics of the brain in individuals with chronic musculoskeletal pain, which is associated with changes in pain processing and patient‐reported outcomes (de Zoete, Chen, et al., 2020). These findings further support previous studies that reported the influence of exercise on pain modulation (Kroll, 2015; Mannion et al., 2012). However, to date, no study has investigated the relationship between the effect of physical exercise and CS outcome measures in individuals with chronic NP.

Recent developments in neuropathology and neurophysiology have found that lactate plays a significant associative role in the development and maintenance of chronic pain (Milligan & Watkins, 2009; Wang et al., 2021). Neuroimaging studies have found that individuals with chronic pain have alterations in the structural properties of the brain and hyperactivation of the anterior cingulate cortex (ACC), both of which are believed to play a major role in the initiation, development and maintenance of chronic pain (de Zoete et al., 2022; Smallwood et al., 2013; Zhuo, 2008). In individuals with chronic pain, the hyperactivation of the ACC results in a high energy demand for lactate by the neurons as an energy source. The high lactate demand, in turn, increases the rate of astrocyte–neuron lactate transfer (ANLT) in the ACC (Suzuki et al., 2011). The increased rate of lactate transfer results in increased expression of p‐ERK, p‐CREB and Fos, which have been identified as requirements for lactate‐transfer‐mediated modulation of chronic pain (Ji & Strichartz, 2004; Ji et al., 1999). A recent study found that the disruption of the ANLT, by blocking glycogenolysis in astrocytes, can disrupt the formation of chronic pain (Wang et al., 2021). Previous studies investigating effects of exercise on chronic musculoskeletal pain reported either a hypoalgesic or hyperalgesic response depending on the modality of exercise performed, the involvement of painful or non‐painful body areas and the duration and intensity of the exercise performed (Ge et al., 2012; Lannersten & Kosek, 2010; Smith et al., 2020; Vaegter & Jones, 2020). Previous studies on aerobic exercise in chronic pain found that 30 min compared with 10 min of aerobic exercise elicited a greater dose response, with a larger effect on exercise‐induced hypoalgesia (EIH) (Hoffman et al., 2004), and there was no difference in the EIH response between 10 and 20 min of aerobic exercise (Vaegter et al., 2014). Although the underlying mechanisms of EIH are not well established, previous research has found that lactate produced in the periphery during exercise can crosses the blood–brain barrier through monocarboxylate transporters and be used as an energy source by neurons (Proia et al., 2016). We hypothesize that by increasing the availability of lactate through exercise, lactate will be transported across the blood–brain barrier via monocarboxylate transporters and taken up by the energy‐deprived neurons, fulfilling the lactate demand and disrupting glycogenolysis in the astrocytes and the ANLT. The disruption of glycogenolysis and ANLT is hypothesized to be crucial to the molecular aetiology of the persistence of pain. Therefore, this molecular bridge provides crucial new insights into the pain‐mitigating benefits of exercise.

Although the evidence indicates that exercise alters central pain modulation, it is not known whether the clinical presentation of CS is associated with the mechanistic effect of exercise on hypoalgesia. Therefore, the primary aim of this study is to investigate the immediate effect of a single bout of physical exercise on CS in individuals with chronic NP. The secondary aim is to investigate the relationship between lactate concentration ([La]), CS and pain sensitivity. With the knowledge that lactate plays an important role in the maintenance of chronic pain, we hypothesize that lactate produced during exercise will have a positive correlation with EIH. Although exercise might induce similar hypoalgesic responses across different chronic pain conditions (Polaski et al., 2019), comparing these effects across different types of chronic pain poses challenges (Rice et al., 2019). Notably, differences in the mechanisms underlying different chronic pain conditions (Bogduk, 2011; Knezevic et al., 2021; Popescu & Lee, 2020) and different management strategies and recovery trajectories have led to different clinical guidelines for different chronic pain conditions (Blanpied et al., 2017; George et al., 2021). To ensure that the findings of this trial are valid and reproducible and optimally inform clinical practice, this study focuses on chronic non‐specific NP alone in order to ensure a homogeneous population.

## 2 METHODS

### 2.1 Funding

This study did not receive any external funding.

### 2.2 Setting and design

**FIGURE 1:**
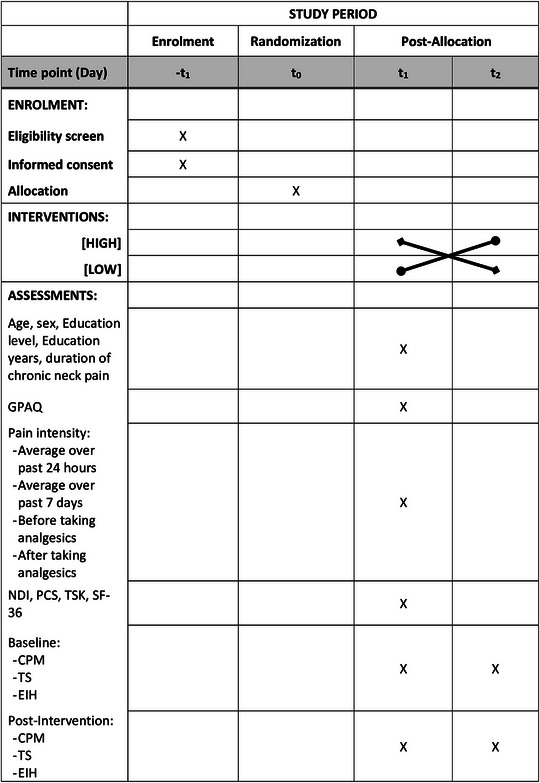
Schedule of enrolment, interventions and assessments. Abbreviations: CPM, conditioned pain modulation; EIH, exercise‐induced hypoalgesia; GPAQ, global physical activity questionnaire; NDI, neck disability index; PCS, pain catastrophizing scale; SF‐36, 36‐item short form survey; ‐t_1_, enrolment week 0; t_0_, allocation; t_1–2_, intervention, baseline and post‐intervention assessments; TS, temporal summation; TSK, Tampa scale of kinesiophobia.

This study will be conducted in accordance with the National Health and Medical Research Council (NHMRC) guidelines and statements and will be performed at The University of Adelaide (SA, Australia). The trial was registered in the Australian New Zealand Clinical Trial Registry (ACTRN12622000642785p) and approved by the Human Research Ethics Committee of The University of Adelaide (HREC‐2022‐082). The study protocol complies with the SPIRIT 2013 recommendations (Standard Protocol Items: Recommendations for International Trials; Supplementary Table S1; Chan et al., 2013). Figure 1 depicts the overall schedule and time commitment for trial participants.

This study is a randomized crossover trial, in which all participants will take part in all interventions, but the order in which they are assigned to the interventions is randomized. Participants will attend the first data collection session, during which baseline questionnaires and physical assessments will be measured, followed by the intervention, and a post‐intervention physical assessment will be measured. There will be a 1‐ to 2‐week washout period before the second data collection session, which is identical to the first session minus the baseline questionnaires, with a different intensity of intervention.

### 2.3 Participants

The sample will consist of 80 adults, who will be enrolled into the study according to the following criteria. Inclusion criteria are as follows: Adults (19–65 years of age); and chronic NP (>12 weeks duration) with and without CS. Exclusion criteria are as follows: Known or suspected serious spinal pathology (e.g., metastatic, inflammatory or infective disease of the spine); confirmed fracture or dislocation at the time of injury; nerve root compromise (at least two of the following symptoms: weakness/reflex changes/sensory loss associated with the same spinal nerve); spinal surgery in the previous 12 months; history or presentation with psychosis, bipolar disorder, organic brain disorder or severe depression; individuals who are currently taking or have a history of taking anti‐depressant or anti‐convulsant medication; and answered ‘yes’ to any of the seven questions on the Physical Activity Readiness Questionnaire (PAR‐Q+) (Warburton et al., 2021).

Participants will be recruited through posters placed on the University campus and at supermarkets, and posts on social media. All potential participants will receive oral and written information, listing the participant's responsibilities, study risk and benefits, prior to prescreening and obtaining informed consent. Participants will be enrolled into the study after obtaining informed consent.

### 2.4 Procedures

Participants who meet the inclusion criteria will be invited to attend two testing sessions: one to perform high‐intensity submaximal aerobic bicycle exercise (HIGH); and a second to complete a low‐intensity submaximal aerobic bicycle exercise (LOW). The order of these two testing sessions will be randomized. Participants will be asked to refrain from analgesics on the day of testing and to abstain from physically demanding activities, including exercise, for 48 h before testing. Participants will also be asked to abstain from nicotine, alcohol and caffeine for 12 h before testing.

**FIGURE 2:**
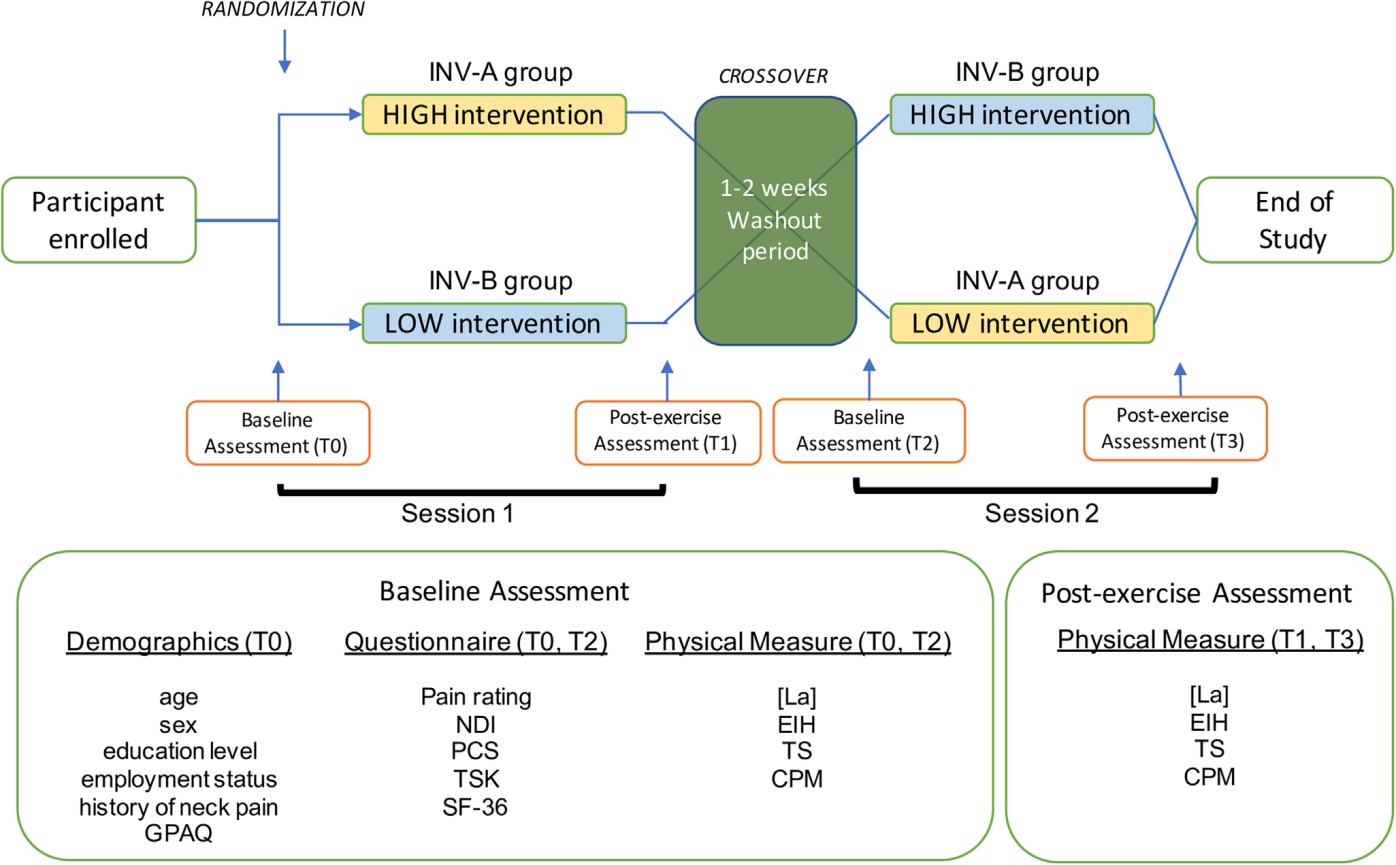
Flowchart of study procedure and the associated assessment conducted.

Participants will be given a detailed explanation about the research study and the requirements of them. Informed consent will be obtained from the participants before collecting any data. Figure 2 depicts the flowchart of study procedure and the assessments conducted at each assessment time point. Participants will be randomized into two main groups, termed the INV‐A group and the INV‐B group, with INV‐A doing HIGH–LOW and INV‐B doing LOW–HIGH. Participants will wait 10 min before commencing the exercise intervention to prevent a carryover effect from the baseline physical assessments influencing exercise. During the waiting time, resting heart rate (HR) and blood pressure will be measured, and appropriate warnings and precautions will be briefed. Participants will then complete the assigned exercise intervention, immediately followed by postexercise assessment. The order of testing will be kept constant within each testing session, both before and after exercise. To prevent a carryover effect, the sessions will be scheduled 1–2 weeks apart.

### 2.5 Randomization

The participants will be enrolled sequentially. After informed consent is obtained, participants will be allocated randomly to one of two groups: INV‐A or INV‐B. Block randomization will be performed with a 1:1 allocation ratio, using the website https://www.sealedenvelope.com/. A table with random numbers generated by the website will be used. Opaque and sealed envelopes containing cards with the name of the intervention group will be used to ensure concealed allocation. A researcher not part of the study will perform the allocation procedure. Owing to the nature of the study trial, blinding of the assessor and participants is not possible.

### 2.6 Data collection

Demographics, including age, sex, education level, duration of chronic NP, physical activity participation level (GPAQ; Cleland et al., 2014) and employment status, will be collected. The outcome assessments consist of questionnaires and central pain processing measurements. Questionnaires will be assessed at the start of each exercise session, and the central pain processing measurements will be performed twice at each session: at baseline; and postexercise.

#### 2.6.1 Questionnaires

##### Numerical pain rating scale

Pain intensity will be measured using the numerical pain rating scale (NPRS), which is a valid and reliable tool (Young et al., 2019). The scale is characterized by a line 10 cm in length, with 11 points (0−10) equally spaced on the line, where ‘0’ indicates no pain, and ‘10’ the worst pain imaginable. Participants will be asked to indicate the average NP intensity over the past 24 h and the past 7 days, by placing an ‘X’ on two separate NPRS lines. The NPRS score of average pain intensity will be measured in centimetres, to the nearest tenths.

##### Neck disability index

Disability will be measured using the neck disability index (NDI) questionnaire (Young et al., 2019), which was validated and found to have a high degree of reliability (Vernon & Mior, 1991). The questionnaire consists of 10 items: pain intensity, personal care, lifting, work, headaches, concentration, sleeping, driving, reading and recreation. Each item is rated on a six‐point scale from zero (no disability) to five (complete disability). The numerical responses for each item are summed for a total score ranging between 0 and 50, with a higher score indicating greater disability. If an item (e.g., driving) does not apply to a participant, the item will be omitted from the questionnaire and a proportion score will be calculated accordingly.

##### Pain catastrophizing scale

The pain catastrophizing scale (PCS) is an instrument used to measure catastrophic thinking related to pain and has been used extensively in clinical practice and research (Sullivan et al., 1995). The scale consists of 13 items, measuring catastrophizing thoughts or feelings accompanying the experience of pain. Participants will be asked to rate on a five‐point scale (ranging from 0 =  not at all to 4  =  all the time), based on past painful experiences, and to indicate the degree to which each of the 13 thoughts or feelings were experienced when in pain. The PCS score ranges from 0 to 52, with higher scores indicating a greater pain catastrophizing.

##### Tampa scale of kinesiophobia‐11

The Tampa scale of kinesiophobia‐11 (TSK‐11) is an abbreviated version of the Tampa scale of kinesiophobia (TSK) questionnaire, used to measure fear of movement/(re)injury (Tkachuk & Harris, 2012). The questionnaire uses four points (ranging from strongly disagree to strongly agree) to assess kinesiophobia based on the model of fear avoidance, fear of work‐related activities, fear of movement and fear of re‐injury. The score for TSK‐11 ranges from 11 to 44, with a higher score indicating greater kinesiophobia.

##### Thirty‐six‐item short form health survey

The 36‐item short form health survey (SF‐36) is a tool frequently used for measurement of health‐related quality of life (Ware, 2000), validated and reliable in measuring self‐reported general health in individuals with chronic NP (de Vries et al., 2015). It comprises of 36 items, covering eight different domains of health: physical functioning; bodily pain; role limitations owing to physical health problems; role limitations owing to personal or emotional problems; emotional well‐being; social functioning; energy/fatigue; and general health perceptions. For the purpose of this study, the score from all domains will be averaged to provide a single SF‐36 score, with a higher score indicating a more favourable health state.

#### 2.6.2 Physical outcome measurements

**FIGURE 3:**
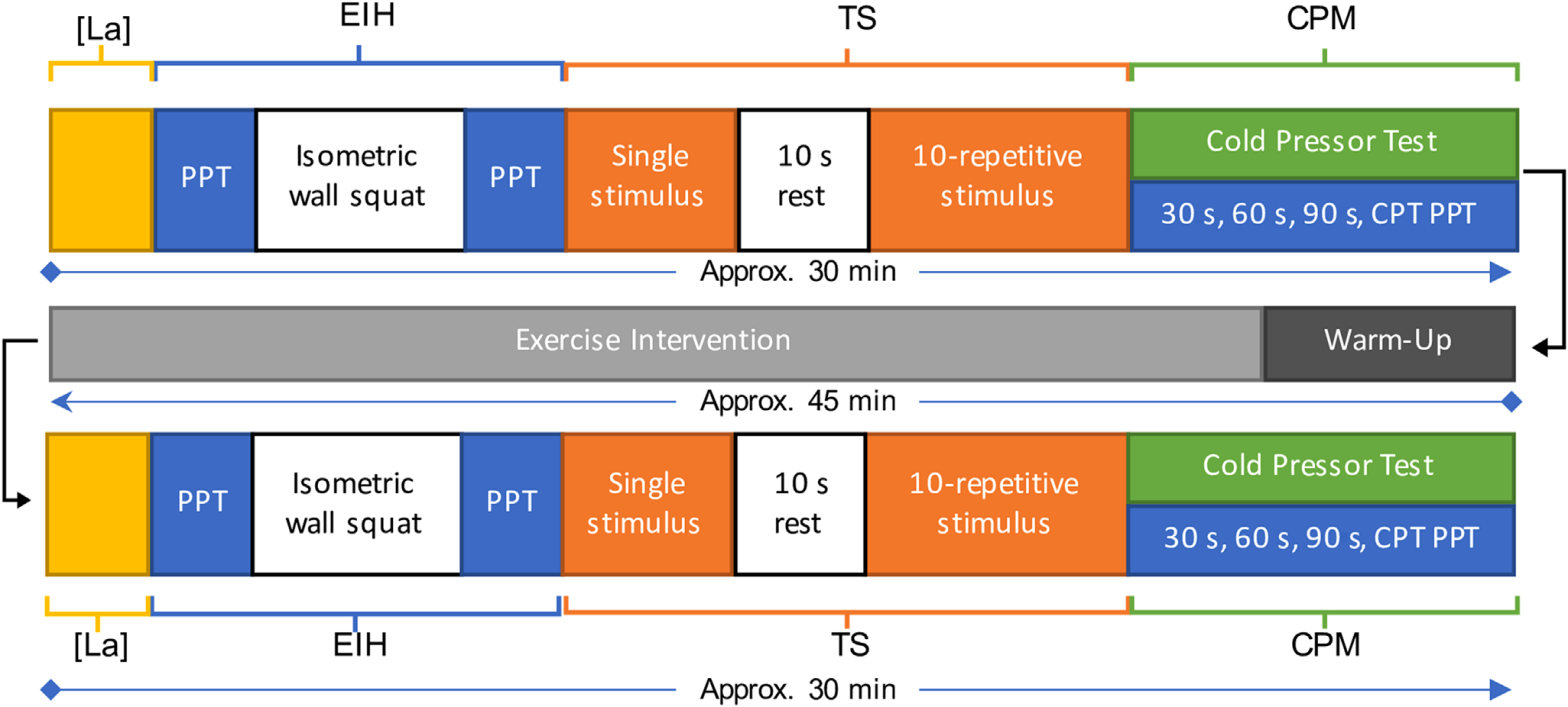
Assessment flowchart at each data collection session. Abbreviations: CPM, conditioned pain modulation assessment; CPT, cold pain threshold; EIH, exercise‐induce hypoalgesia assessment; [La], blood lactate concentration assessment; PPT, pain pressure threshold; TS, temporal summation assessment.

Figure 3 depicts the flowchart of assessment conducted at each data collection session.

##### Primary outcome measures

###### Exercise‐induced hypoalgesia

Exercise‐induced hypoalgesia will be assessed through pain pressure threshold (PPT) measurement, followed by a fatigue‐inducing exercise stimulus, and lastly, the same PPT measurements. The PPT will be measured using Echo Wireless Algometer (JTECH Medical, UT, USA). The PPT will be measured at two sites; (1) over the C5/C6 spinous process in the cervical spine; and (2) over the muscle belly of the left tibialis anterior. To ensure accurate measurement, an ‘x’ will be marked on the testing site with a marker during baseline assessment, and the same site will be used for postexercise PPT assessment. Pressure will be applied on the testing site at a rate of ∼5 N/s. Participants will be asked to state verbally when the sensation changes from ‘comfortable’ pressure to ‘unpleasant’ pain sensation. Triplicate reading, with a 30 s interstimulus interval, will be taken at each site, and the mean values will be used for analysis. The PPT measurements will be taken before and after the fatigue‐inducing exercise stimulus. The fatigue‐inducing exercise stimulus will be an isometric wall‐squat. Participants will be asked to maintain a wall‐squat position until fatigue or for a maximum of 3 min.

###### Temporal summation

Temporal summation (TS) will be induced using a 256 mN punctate needle stimulator or ‘PinPrick’ (MRC Systems, Heidelberg, Germany) as the noxious stimuli, at two sites: (1) over the C5/C6 spinous process in the cervical spine; and (2) dorsal surface of the dominant hand between the second and third metacarpals. An ‘x’ will be marked at the testing site to ensure accuracy for baseline and postexercise assessment. In the test, a single stimulus will be applied initially, and participants will be instructed to rate the pain intensity verbally. Then after a period of 10 s, a series of 10 identical punctate stimuli will be applied at a frequency of 1 Hz, which has been shown to be sufficient to induce TS. The participant will state verbally the pain rating immediately following the last stimulus. A printed NPRS, labelled with digits from 0 to 10, with ‘0’ indicating ‘no pain’ and ‘10’ being ‘extremely painful’, will be displayed in front of the participant.

###### Conditioned pain modulation

Conditioned pain modulation (CPM) will be measured using the PPT as the test stimulus and the cold pressor test as the conditioning stimulus. The PPT will be measured using Echo Wireless Algometer (JTECH Medical, UT, USA). The baseline PPT (PPTbase) is obtained from EIH assessment. The participants will be instructed to immerse their non‐dominant hand in an ice‐bath (up to the wrist) and will be motivated through verbal encouragement to maintain immersion for the duration of the defined period of 120 s. Participants will be instructed to state verbally when he/she begins to feel pain. The cold pain threshold is the time (in seconds) between the immersion and the verbal confirmation of pain sensation. The cold pressor pain tolerance is the total time (in seconds) of immersion. A single PPT will be measured over the previously marked site on the tibialis anterior at 30, 60 and 90 s and at the time of cold pain threshold occurring during immersion.

##### Secondary outcome measures

###### Lactate concentration

The [La] will be analysed using the Lactate Scout 4 lactate analyser (SensLab, Leipzig, Germany). A drop of blood, obtained using a single‐use lancing device on the fingertip, will be required for analysis. To ensure accuracy in measurement, the manufacturer's protocol guidelines will be followed.

### 2.7 Exercise interventions

All assessment sessions will be conducted at The University of Adelaide.

#### 2.7.1 HIGH intervention

Participants will put on the Polar HR monitor chest strap (Polar H10; Polar Electro Oy, Kempele, Finland). The HR monitor will be connected to a bluetooth‐enabled device, which will display the HR. After measuring the resting HR and blood pressure, [La] will be measured. The rating of perceived exertion (RPE) scale will be explained to the participants. The RPE will be determined using Borg 6–20 point‐category scale (Williams, 2017). The participant's 75% of age‐predicted maximum HR [i.e., 0.75 × (220 − age in years)] will be calculated; this will be used as the target HR during exercise.

Participants will then go onto the stationary bicycle, with the seat height adjusted for optimal cycling posture. Participants will use the same bicycle, with the same adjustment made, on both sessions. After a quick instruction briefing to ensure safety, participants will then complete a standardized, submaximal bicycle aerobic exercise protocol, based on the aerobic power index (API) protocol. The API protocol has been found to be a safe and reliable submaximal exercise test for individuals with various clinical conditions (Furzer et al., 2012; Wallman et al., 2003), including chronic NP (Ickmans et al., 2017; Smith et al., 2017, 2020). The participants will start to cycle at 60–70 rpm at level 1 resistance, and the resistance will be increased by one or two levels every minute until attainment of the target HR. The increment of resistance level will be controlled by the researcher and will be based on the participant's HR response. The participants will then continue to cycle at this power output for 30 min. Heart rate and RPE will be recorded each minute during the increase in power output, then once every 3 min until the end of the exercise session.

#### 2.7.2 LOW intervention

The LOW intervention will follow the same procedure as HIGH, except for the intensity of the exercise. The participant's target HR will be 50% of age‐predicted maximum HR.

### 2.8 Sample size

For this clinical trial, the sample size is calculated based on PPT. Calculations for an appropriate sample size were performed with G*Power (v.3.1.9.6; Dusseldorf, Germany) (Faul et al., 2007). Given that there are no data on aerobic exercise testing with similar intervention groupings in chronic NP, the a priori power analysis was conducted based on data from an exercise intervention study with similar intervention grouping (Taimela et al., 2000). The sample size of this study is calculated based on the difference in mean PPTs (25.0 ± 19.7; 42.1 ± 27.2) between two different exercise interventions, a desired two‐tailed α = 0.05, β = 0.20, and an allocation ratio of 1:1. The calculations revealed that 64 participants would be sufficient. However, to allow a loss to follow‐up of 20% and taking a conservative approach, a total of 80 participants with chronic NP will be recruited.

### 2.9 Statistical analysis

All data will be analysed via scatterplots, boxplots and Kolmogorov–Smirnov statistics to evaluate normality. Normally distributed data will be reported as the means and 95% confidence interval. Other data will be reported as the median and interquartile range. Parametric tests will be used for normally distributed data, and non‐parametric tests for non‐normal distributed data, to analyse between‐group differences for physical outcome measures and baseline questionnaires and physical measures.

To test for carryover effects, the sum of the measured values in the sessions for each patient will be calculated and compared across the two groups with Student's unpaired *t*‐test (Wellek & Blettner, 2012). A *P*‐value of >0.05 indicates no carryover effects, and the ‘washout’ duration is sufficient. To determine the statistical difference between HIGH and LOW, the within‐subject difference in outcome parameters between both sessions will be calculated using Student's unpaired *t*‐test. If there is a statistical significance, a regression model will be conducted to evaluate the effect of exercise intensity (HIGH vs. LOW) on CS measures. The fixed effect of time (baseline and postexercise), exercise intensity and study group will be included in the model, with age as a covariate. If no statistical difference is detected, repeated‐measures ANOVA will be performed to assess significant differences between both groups; the within‐subject factor will be defined as ‘time’, with ‘group’ as a between‐subject factor. The significance level will be adjusted by the number of comparisons using Bonferroni correction.

Multivariate regression analysis will be used to analyse the association between [La] and EIH. In order to examine the relationship between individual measures of CS (EIH, CPM and TS), separate regressions will be conducted. Models will be adjusted for age, sex, education level, physical activity level, NDI, PCS and TSK. Statistical significance is set at *P* < 0.05.

## 3 DATA COLLECTION AND QUALITY ASSURANCE

Study data will be collected using standardized data collection forms and entered electronically into the study database as soon as possible and at least weekly. Paper forms will be used to document informed consent, adverse events and protocol non‐compliances. Participant information with personal identifiers will be stored in paper‐based form in a locked filing cabinet in a secure and locked office. All data entered electronically into the study database will be only de‐identified data. The study database will be stored on a password‐protected secure sever. To ensure accuracy and completeness, study data will be reviewed routinely by study team members. Standard University's data management policy (data planning, storage, retention and planning, access and re‐use, disposal and resolution of disagreement) (Research Data and Primary Materials Policy, version D2021/156953) will be followed to ensure accurate, reliable and consistent data collection.

## 4 DATA MANAGEMENT

A research data management plan has been registered with the University. Study data management activities, including data entry and validation, data coding and cleaning, database quality control, adverse event reporting and preparation of the final study database will be conducted by the study team members. The study team will be responsible for maintaining and storing securely the complete, accurate and current study records throughout the study, including signed informed consent. Privacy of all participants will be protected, and all data concerning people will remain confidential in order that their identities and any type of identifying information is protected. All participants enrolled in the study will be assigned a study code. All data collected will be labelled with the code from the first session of data collection. A list of codes and corresponding participant identifiers will be kept separate from coded data and available only to the researchers. Data queries will be resolved as soon as possible, and missing data accounted for in statistical analysis. All data management activities will be incompliance with The University of Adelaide (The University of Adelaide, 2021) and the National Health and Medical Research Council (NHMRC) policy and guidelines (National Health & Medical Research Council, Australian Research Council & Universities, Australia). After publication of all study results, the data underlying the findings will be made available in a self‐service, online public data repository.

## 5 ETHICS AND DISSEMINATION

### 5.1 Ethics approval and consent

The study will be conducted in compliance with the NHMRC National Statement on Ethical Conduct in Research Involving Humans 2007 (updated 2018) (National Health & Medical Research Council, Australian Research Council & Universities, Australia). The protocol and other relevant study document were approved by The University of Adelaide's Human Research Ethics Committee (HREC‐2022‐082) and have been registered in the Australian New Zealand Clinical Trial Registry (ACTRN12622000642785p). Participants will be recruited from the general public, through recruitment fliers, websites and social media. Potential participants will be given the information about the study and will be given adequate time to ask questions. Potential participants will then be screened for eligibility. Written informed consent will be obtained by study team members before enrolment and any data collection.

### 5.2 Safety

All participants will be screened before enrolment using the PAR‐Q+ questionnaire to assess whether a participant is physically ready and safe to participate in the study. Stringent coronavirus disease 2019 precautions will be enforced according to university and government guidelines throughout the study. Baseline resting HR and blood pressure will be measured at each session, before exercise, to ensure the participant's safety. All data collection sessions will be supervised by an experienced exercise physiologist and overseen by a senior physiotherapy academic. All physical outcome measures and the exercise protocol have been found to be safe for the study population and have been used widely in various studies (Ickmans et al., 2017; Smith et al., 2017; Vaegter et al., 2016). A risk assessment and management plan has been developed with the University's Health, Safety and Wellbeing Team to eliminate or manage the likelihood and consequences of adverse events occurring. All adverse events will be assessed and managed in accordance with standard clinical practice at the study site and documented by the study team. All serious adverse events will be reported to the principal investigator and will be reported as required by the HREC approval process, and the appropriate steps will be taken, following the University's policy and guidelines. All adverse events will be recorded according to the registered data management plan.

### 5.3 Possible risks

There are potential risks to study participation. This is a randomized crossover trial investigating the effect of a single bout of two different aerobic exercise intensities on individuals with chronic NP. It is possible that the pain intensity and sensitivity might increase after exercise. With all exercise interventions there is increased risk of adverse events, such as cardiorespiratory events and musculoskeletal injuries. With prescreening conducted before the start of the intervention, the risks of adverse events are mitigated, and risks to participants are not anticipated to differ from ordinary risks of participating in any physical activities. Furthermore, the study has been developed carefully and designed pragmatically, with inclusion and exclusion criteria, to allow results to be as generalizable as possible without putting participants with severe conditions at risk, and a robust safety protocol will be in place. Participants with health conditions prohibiting them from performing exercise are excluded from the study. All potential participants will be given a ‘participant information sheet’ listing all study information, including potential risks, and all queries will also be answered before obtaining informed consent. All exercise sessions will be supervised by an experienced exercise physiologist and overseen by a senior physiotherapy academic.

### 5.4 Dissemination

We plan to disseminate study results in peer‐reviewed journals and international conferences targeting those involved in research and/or clinical care of individuals with chronic NP.

## 6 DISCUSSION

There are numerous research studies and systematic reviews on the effects of exercise on chronic NP, covering a wide variety of modalities of exercise (Bertozzi et al., 2013; de Zoete et al., 2019; de Zoete, Armfield, et al., 2020; Gross et al., 2015; Price et al., 2020; Sterling et al., 2019). However, systematic reviews have found that the effectiveness of exercise interventions for improvement of pain intensity and disability of chronic NP is modest at best (de Zoete et al., 2019; Price et al., 2020; Sterling et al., 2019), with no high‐quality evidence (Gross et al., 2015). Numerous studies have suggested that the presence of CS could be a factor mediating the effectiveness of exercise (Hansen et al., 2020; Kroll, 2015; Mannion et al., 2012). The difference in the presence of CS in chronic NP patients might be one of the factors contributing to the overall modest effect of exercise. However, to date, no study has investigated the relationship between the presence of CS and the mechanistic effect of exercise on hypoalgesia. Therefore, we aim to investigate the effect of two different intensities (HIGH vs. LOW) of a single bout of submaximal aerobic exercise on measures of CS in individuals with chronic NP.

We will also conduct a new investigation into the relationship of lactate, produced during exercise, with the underlying mechanism of EIH. Recent studies have found that lactate plays a role in maintaining chronic pain through its effect on the CNS, by affecting specific neurophysiological pathways within the brain (Wang et al., 2021). Previous studies of the effect of aerobic exercise on chronic pain found that 30 min compared 10 min of aerobic exercise elicited a greater dose response, with a larger effect on EIH (Hoffman et al., 2004), and there was no difference in the EIH response between 10 and 20 min of aerobic exercise (Vaegter et al., 2014). Therefore, we hypothesize that the [La] produced during exercise could play a role in the hypoalgesic effect of exercise.

The study design allows investigation of the mechanistic effect of a single bout of exercise on outcome measures of CS. One limitation of this study is the inability to assess effects of the exercise intervention, hence it is not directly translatable into clinical settings. Ideally, a longer intervention duration (e.g., 4–6 weeks of exercise training) could provide insights into the effect of the exercise intervention prescribed on patient‐reported outcomes (i.e., pain rating and NDI). However, previous studies have found that EIH can be induced after a single bout of exercise, and the effect of EIH can last ≤30 min postexercise (Rice et al., 2019). Combined with the new investigation on lactate, we can gain insights on the mechanistic effect of a single bout of submaximal aerobic exercise on CS. In addition, we will gain knowledge of the immediate effects of exercise in individuals with chronic NP, which could, indirectly, be useful in clinical practice.

One major strength of this study is the sample size. Previous studies that included PPT, TS or CPM measures had a sample size of ≤61 participants, with only one study having 96 participants (Vaegter et al., 2021). However, in this larger study (Vaegter et al., 2021), the exercise intervention prescribed was a 6 min walk test. Therefore, with the sample size of 80, the present study has a much larger sample size than other similar studies.

The outcome of this study has the potential to add important information to the current treatment guidelines and influence the exercise prescription and decision‐making of health‐care professionals. It will provide new insights into the mechanistic effect of exercise on CS in individuals with chronic NP and neurophysiological understanding of chronic pain. Results from this study have the potential to be translated for use in other chronic musculoskeletal pain conditions.

## AUTHOR CONTRIBUTIONS

Conception or design of the work: Kexun Chen, Mark Hutchinson, Paul Rolan and Rutger de Zoete. Acquisition, analysis or interpretation of data for the work: Kexun Chen and Rutger de Zoete. Drafting of the work: Kexun Chen, Mark Hutchinson, Paul Rolan and Rutger de Zoete. All persons designated as authors qualify for authorship, and all those who qualify for authorship are listed. All authors approved the final version of the manuscript and agree to be accountable for all aspects of the work in ensuring that questions related to the accuracy or integrity of any part of the work are appropriately investigated and resolved. All persons designated as authors qualify for authorship, and all those who qualify for authorship are listed.

## CONFLICT OF INTEREST

None declared.

## FUNDING INFORMATION

None.

## Supporting information

Supplementary Table S1. SPIRIT 2013 Checklist
